# Supplementary material for: Effect of A-Site Nonstoichiometry on Defect Chemistry and Electrical Conductivity of Undoped and Y-Doped SrZrO3
Source: Materials (Basel). 2019 Apr 17;12(8):1258. doi: 10.3390/ma12081258 (PMC6514593; doi:10.3390/ma12081258)
Supplement: Supplementary file 1 [file materials-12-01258-s001.pdf]

Supplementary Materials: Effect of A-Site Nonstoichiometry on Defect Chemistry and Electrical Conductivity of Undoped and Y-Doped $SrZrO_3$

Liliya Dunyushkina *, Adelya Khaliullina, Anastasia Meshcherskikh, Alexander Pankratov and Denis Osinkin

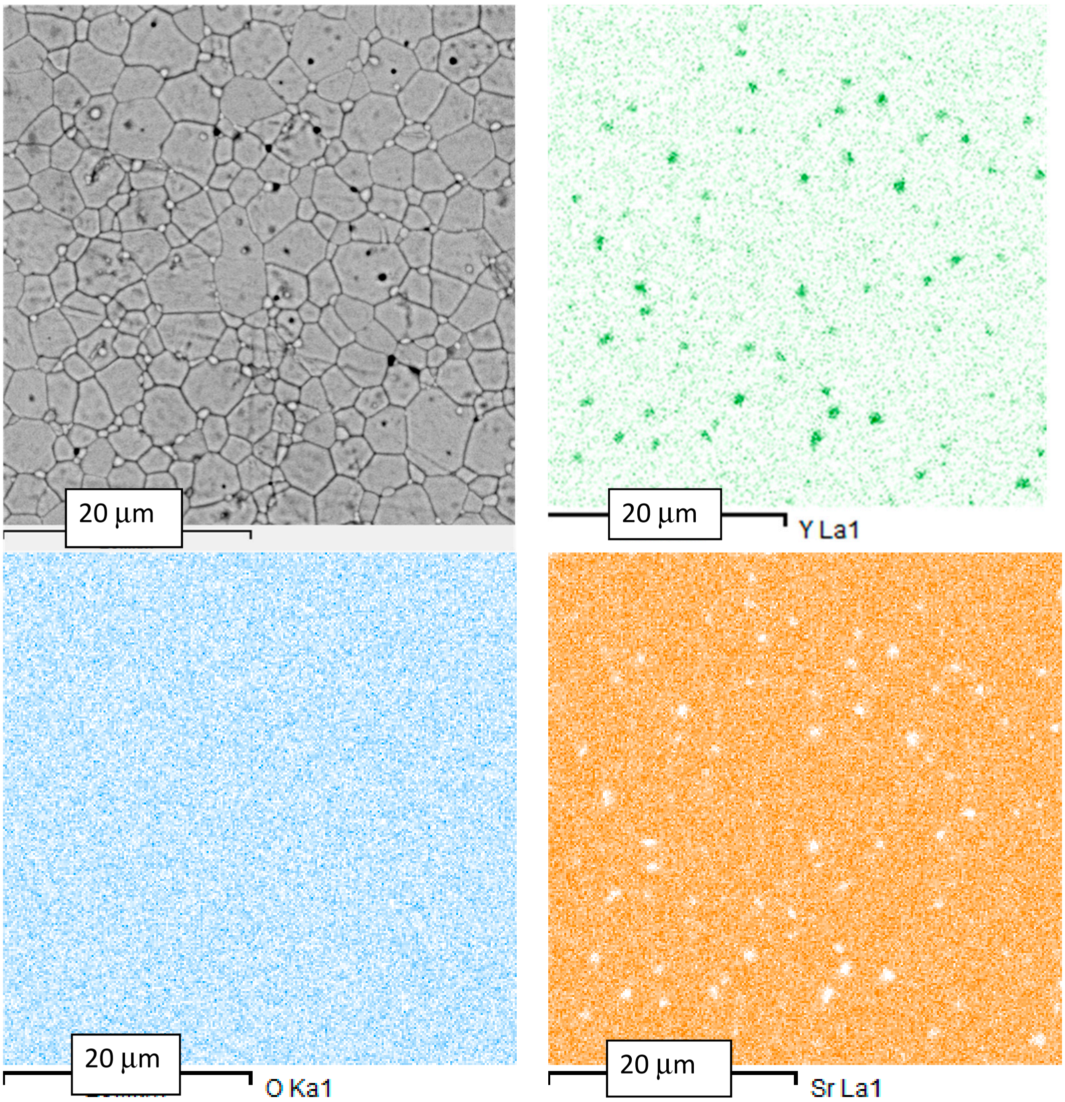

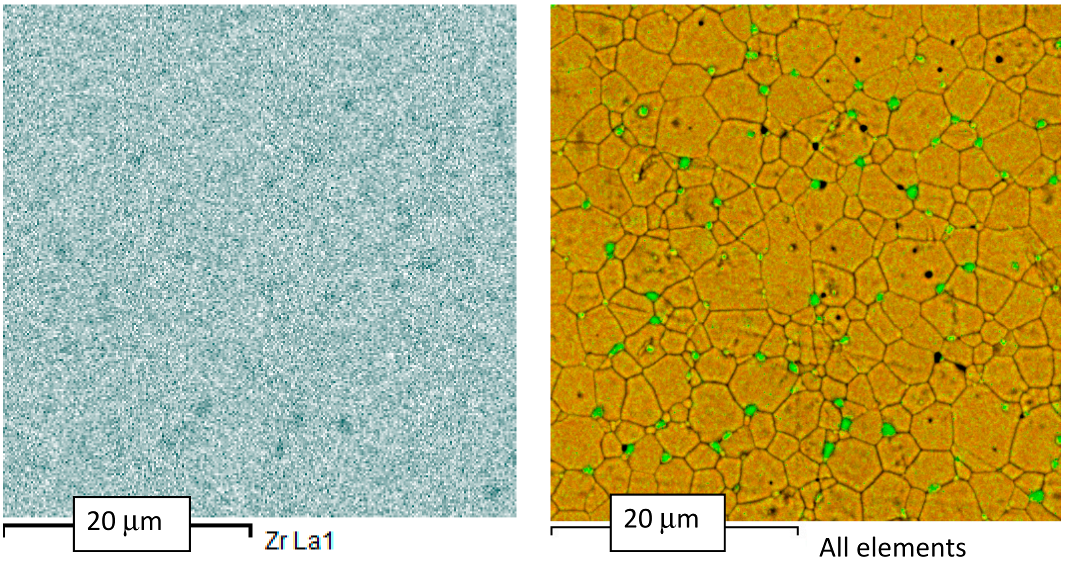

Figure S1. SEM image and EDX mapping of the elements on the surface of SZY0.94 after polishing and thermal etching at 1400 °C.

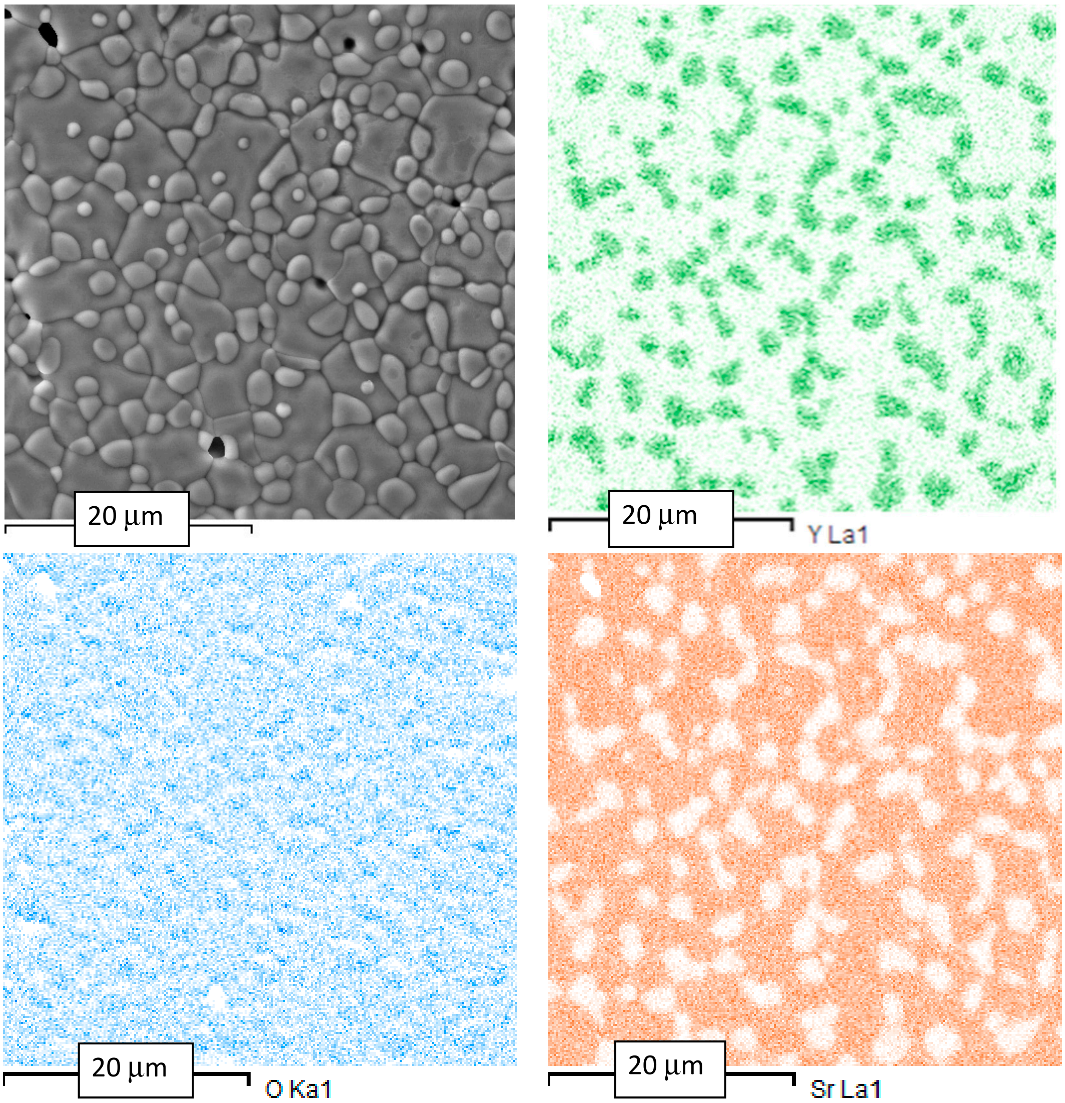

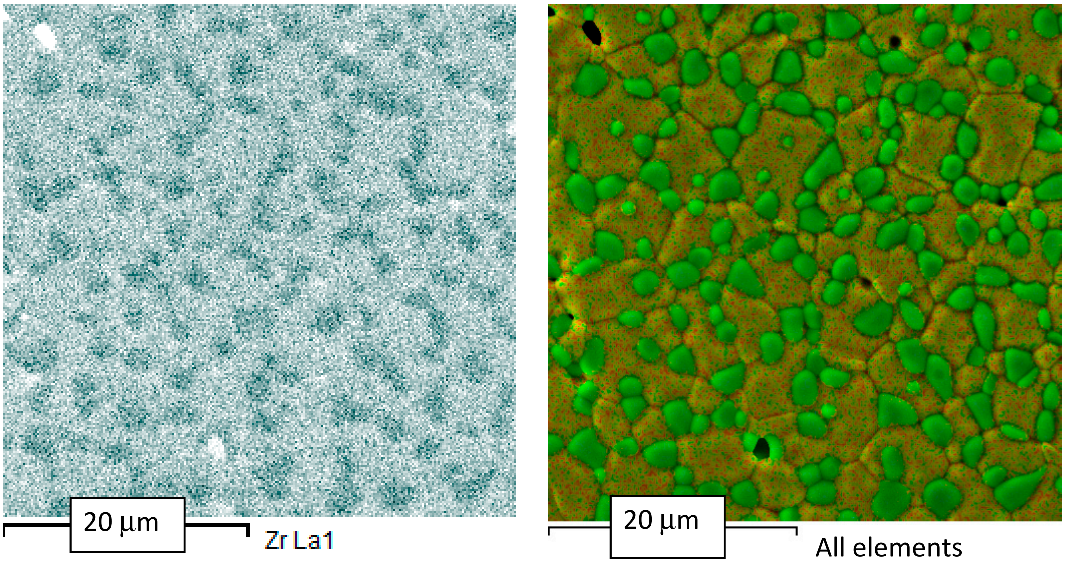

Figure S2. SEM image and EDX mapping of the elements on the surface of SZY0.94 after polishing and thermal etching at 1650 °C.

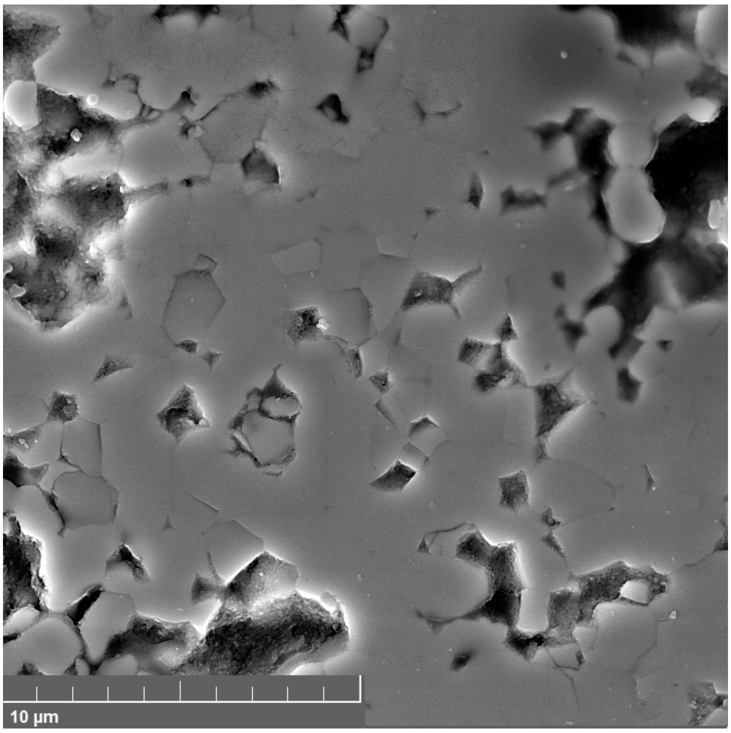

Figure S3. SEM image of as-polished cross-section of SZY1.00.
